# Simultaneous retrograde venous and anterograde arterial bullet embolism: a case report

**DOI:** 10.1186/s13256-022-03414-7

**Published:** 2022-05-22

**Authors:** Ahmad Hosseinzadeh, Mohammad Moeini Farsani, Mohamad Mahdi Mahmoudi, Ahmadreza Hekmatnia, Meghdad Ghasemi Gorji, Majid Asnaashari, Hamed Ghoddusi Johari, Reza Shahriarirad

**Affiliations:** 1 Department of Vascular Surgery, Shiraz University of Medical Science, Shiraz, Iran; 2 Thoracic and Vascular Surgery Research Center, Shiraz University of Medical Sciences, Shiraz, Iran; 3 Department of Surgery, Faculty of Medicine, Bushehr University of Medical Sciences, Bushehr, Iran

**Keywords:** Gunshot, Embolism, Heart, Femoral vein, Femoral artery, Venous system

## Abstract

**Background:**

Bullet embolus is a rare condition following gunshot injuries and represents a clinical challenge regarding both diagnosis and management.

**Case presentation:**

We report the case of a 35-year-old Iranian (Middle-Eastern) male patient with a shotgun injury to both buttocks, which traveled to the heart and the popliteal area through the femoral vein and superficial femoral artery, respectively. Surgical intervention was applied for the popliteal pellet, and the patient was discharged without further complications.

**Conclusion:**

Although bullet emboli can be a clinical challenge, with the advent of modern procedures, removal has become safer. X-ray, computed tomography, and transthoracic and/or transesophageal echocardiography may be used as adjuncts to help establish the diagnosis.

## Background

Usually, when a bullet penetrates the human body, two scenarios occur. Either it goes through the body by traveling in a straight line and exiting the body through the other side, or it dissipates its kinetic energy and stops inside the soft tissues. On rare occasions, the bullet penetrates the wall of an arterial or venous vessel and may even enter the vascular system. Bullet emboli rarely occur on these occasions [1], and there is little expertise with them at any institution, thus representing a challenging entity. Retrieval (by either open surgery or endovascular intervention) and observation are two options. Since its discovery in 1917, surgical intervention has remained the mainstay of therapy [2]. Herein, we report a case of gunshot injury to the buttocks that resulted in both retrograde and anterograde embolism of the bullet pellet, ending up in the heart and popliteal.

## Case presentation

**Fig. 1:**
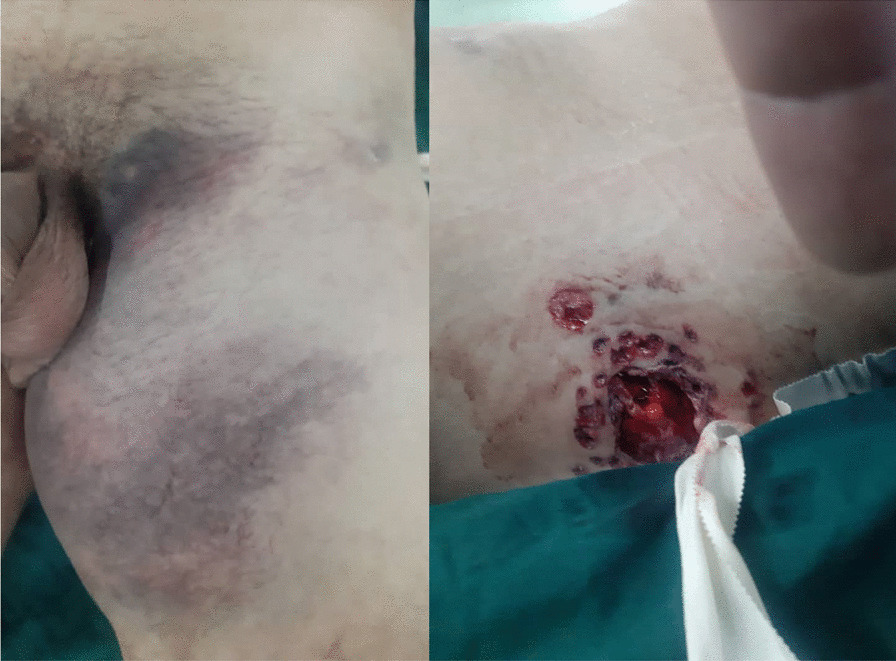
Shotgun injury to the buttock, demonstrating bruising and hematoma along with the point of entry

The patient is a 30-year-old Iranian (Middle-Eastern) male who was previously in a relatively healthy state with no past medical history or addiction and was transferred to our emergency department with two shotgun injuries to both buttocks from close range (1 m). The patient was well oriented (GSC 15) with vital signs of blood pressure 100/70, respiratory rate 20, temperature 36.7 °C, and O_2_ saturation of 96%. On examination, the distal pulse of his left limb could not be detected and was cold, with a hematoma at the injury site. Color Doppler sonography of lower limbs demonstrated weak arterial flow in distal arteries. Abdominal fast sonography was unremarkable. The injury to the right buttocks was not significant, causing only mild bruising and hematoma (Fig. 1).

He received pethidine for his pain along with 1 g cephazolin for infection prophylaxis, and also was hydrated with 2 L normal saline due to low blood pressure. Initial laboratory evaluation demonstrated low hemoglobin levels (10 g/dl), for which he received two bags of packed cells, with a platelet count of 107,000. Coagulation tests were normal.

**Fig. 2:**
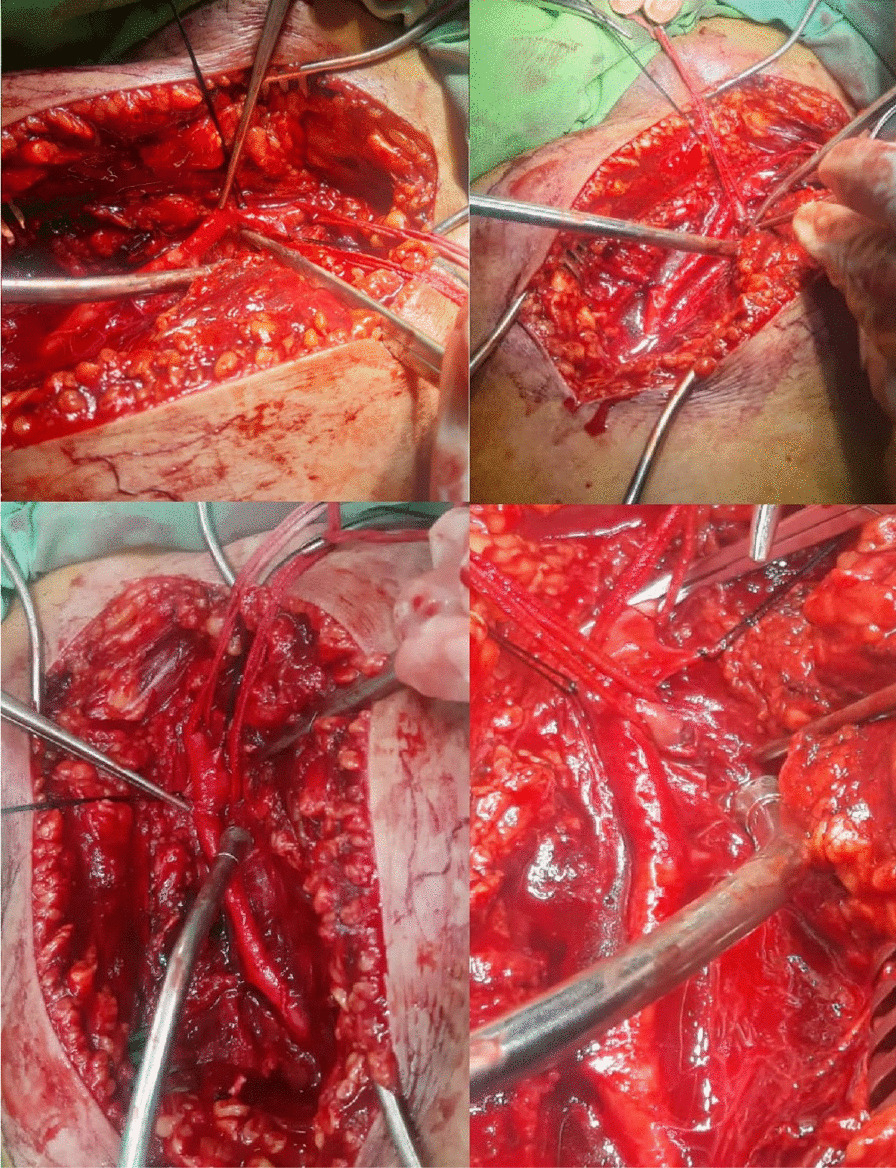
The deep femoral artery and posterior superficial femoral artery injury due to bullet

**Fig. 3:**
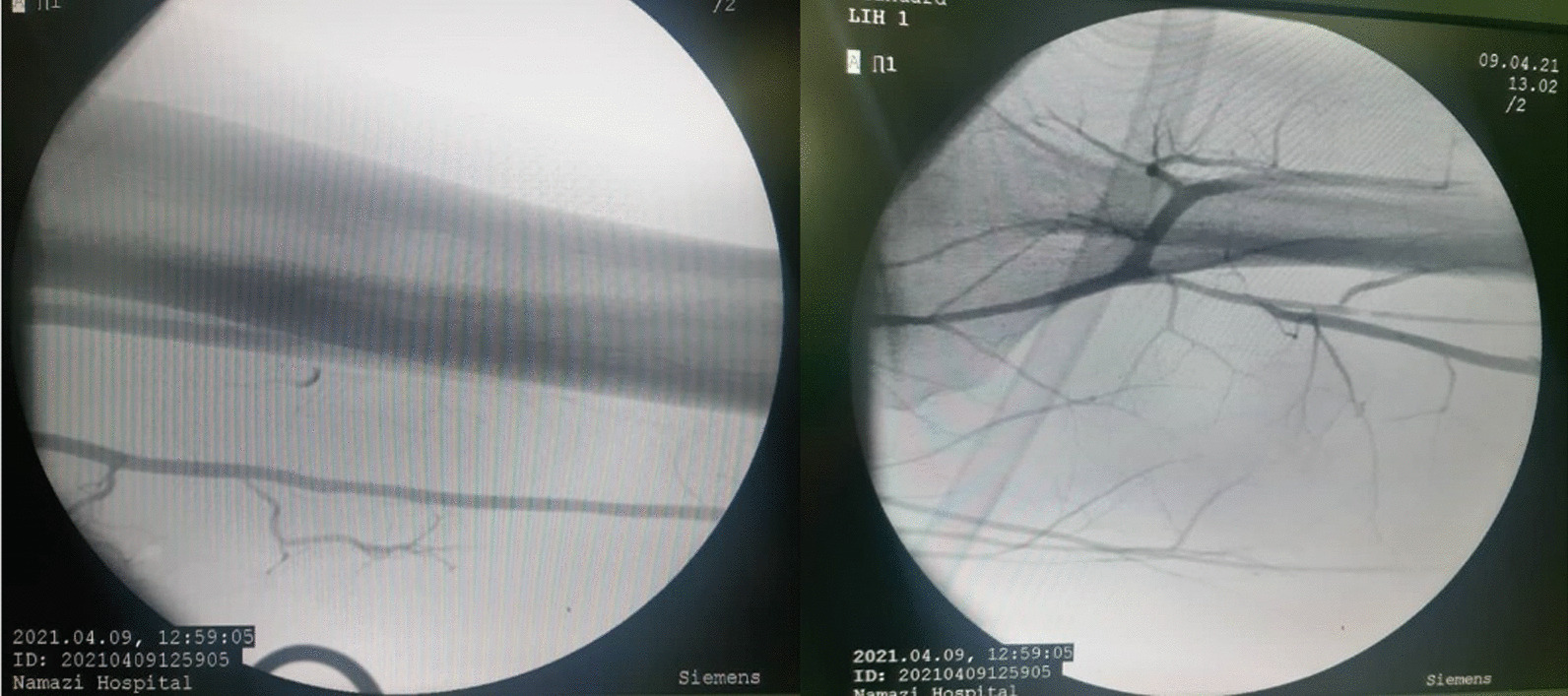
Superficial femoral artery and trifurcation of the popliteal artery after Fogarty, demonstrating no bullet pellets at the lower extremities

**Fig. 4:**
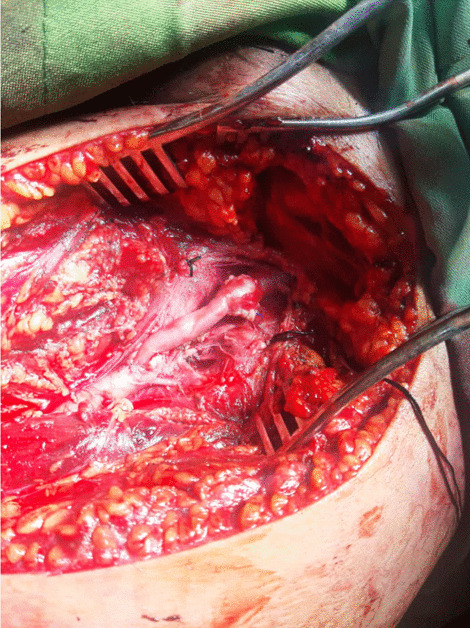
Superficial femoral artery with saphenous graft anastomosis

The patient was scheduled for operation, in which a longitudinal incision was made in the left groin, and the left common femoral artery and vein were explored. On evaluation of the entry point, on the left side, the bullets cut off the origin of the deep femoral origin and the femoral vein, in which avulsion of the proximal end of the left deep femoral artery and partial tearing of the proximal left superficial femoral artery (SFA) (intimal injury) were detected (Fig.  2). The deep femoral artery was ligated with a silk 2-0, and the right thigh great saphenous vein was harvested and dilated. After infusion of systemic heparin, the injured part of the SFA was excised, and the proximal and distal parts of the SFA and the popliteal artery were catheterized with Fogarty 4F, followed by thrombectomy, in which the pellet was removed with the Fogarty. It was observed that the bullet had subsequently entered the SFA and traveled to the trifurcation of popliteal deep peroneal. Digital angiography of the left lower extremity was performed, demonstrating the trifurcation of posterior tibialis, anterior tibialis, and peroneal artery to be patent (Fig.  3). There were no signs of external injury in the popliteal area. Subsequently, interposition graft with the great saphenous vein was done with proximal and distal anastomoses, with Prolene 5-0 in a continuous manner. The flow was restarted in the limb, hemostasis was obtained, and a hemovac drain was inserted. The wound was repaired in layers with Vicryl 2-0 and Nylon 2-0, along with applying a dressing and a long leg slab (Fig. 4).

**Fig. 5:**
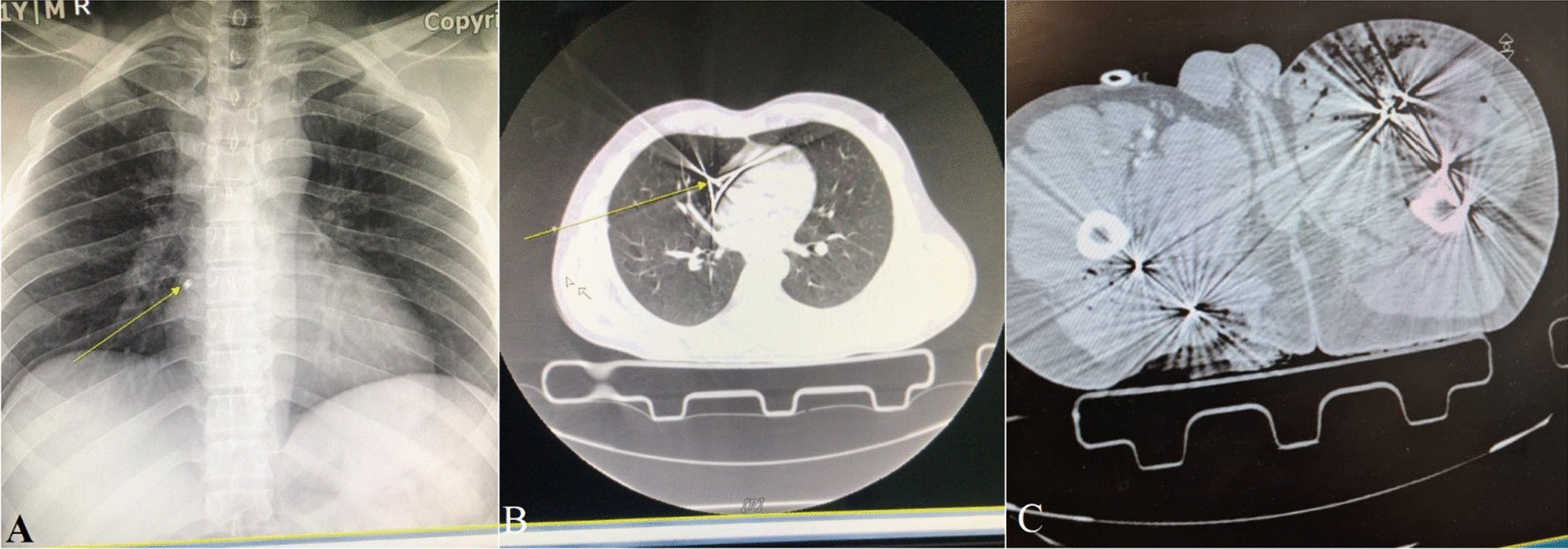
Radiological evaluation of the patient. (**A**) Chest X-ray at 1 day after surgery, demonstrating bullet pellet in the right atrium (yellow indicator). **B** Chest computed tomography scan demonstrating bullet in the branch of the right pulmonary artery (yellow indicator). **C** Lower-extremity computed tomography scan demonstrating bilateral shot gun injury to both gluteal and posterior thighs

After the operation, the patient was stable, with a hematoma present in the left groin. On reevaluating the patient’s chest radiography and computed tomography (CT) scan, it was noticed that one of the pellets (3 mm × 5 mm) had traveled to the heart (branch of the right pulmonary artery), probably through the left femoral vein and causing a local hematoma (Fig. 5). Echocardiography evaluation was unremarkable with ejection fraction of 60% and no signs of foreign objects in the heart. After consultation with a cardiothoracic surgeon, the patient was discharged in good condition, with no cardiovascular complications during his follow-up. The patient remained relatively well up to 1 year after surgery and developed no further complications or complaints. Chest X-ray was requested for the patient during his follow-up, which showed no changes compared with the postoperative image. The patient is now in his 18th month of follow-up, in which the gluteal wounds have healed and he is receiving a therapeutic dose of rivaroxaban (20 mg/day) with no further complications or changes in the particle positioning.

## Discussion and conclusions

We report a unique case of bullet pellet emboli, which traveled to the heart and the popliteal area. The pellet was missed during the initial evaluation of the patient and was also not detected during echocardiography. Diagnosis of bullet emboli might be a challenging issue. Based on studies of war victim, 0.3% and 1.1% incidence of bullet emboli from penetrating vascular trauma patients have been reported in Vietnam and the Afghanistan and Iraq wars, respectively [1, 3] Shannon *et al.* [4] investigated 126 cases of venous bullet emboli in 1987 and found that 48% lodged in the right heart, 36% continued into the pulmonary circulation, and 16% entered the peripheral/central veins. They also observed that missile emboli were not removed in 51 initially asymptomatic individuals. Yoon *et al.* [5] in a systematic review in 2018 also reported that 64.7% of venous emboli go to the right heart.

Bullet emboli are classified as arterial (75%), venous (20%), or paradoxical (5%) [6]. While arterial emboli are usually treated by removing them as soon as possible to avoid distal ischemia, the management of venous cardiac emboli has very limited evidence. However, shrapnel migration via the thoracic area to the inferior vena cava (IVC), heart, and/or pulmonary arterial tree is a cause for concern and may even be asymptomatic, similar to our case. Bertoldo *et al.* stated that 70% of patients with venous bullet emboli are reportedly asymptomatic [6]. Furthermore, Yoon *et al.* [5] in a systematic review regarding venous bullet emboli that traverse to the heart and great vessels reported that most patients (88.7%) were asymptomatic from the bullet emboli to the thoracic cavity. Among the symptomatic patients, 11.3% demonstrated symptoms directly associated with the cardiac venous emboli, such as dyspnea, chest pain, or fever.

It is still debatable whether or not a foreign body that embolizes in venous vessels should be removed. Migration into the vascular system often occurs due to fragmentation or small-caliber bullets [7]. The retrograde migration in blood flow can be caused by the patient position (upright) at the moment of injury and/or positional changes of the body, gravity, respiratory movements, reversal of blood flow by coughing or with the Valsalva maneuver, the presence of a vessel valve, and the size, weight, or shape of the pellet [6].

Many management options have been reported in literature, from cardiac bypass and open-heart surgery [8, 9]. Until more recently, selective observation and endovascular interventions were practiced [10, 11]. Those patients with cardiac emboli that become symptomatic should have the foreign body removed [4]. Some authors advise not retrieving the bullet if it is smaller than 5 mm in diameter, smooth in appearance, uncontaminated, and firmly lodged, and the patient is hemodynamically stable without arrhythmia or valvular dysfunction [1, 4, 5, 12]. Nevertheless, patients with cardiac emboli who become symptomatic should have the foreign body removed [4]. In our case, since the patient was hemodynamically stable, no interventions were applied and the patient was discharged with routine follow-up.

## Conclusions

Bullet emboli can be a clinical challenge, but with the advent of modern procedures, removal has become safer. X-ray, CT, and transthoracic and/or transesophageal echocardiography may be used as adjuncts to help establish the diagnosis, although the strategy must be tailored to the patient’s unique clinical situation.

## Data Availability

All data regarding this study has been reported in the manuscript. Please contact the corresponding author if you are interested in any further information.

## Abbreviations

CT: Computed tomography
SFA: Superficial femoral artery
